# Sequencing and analysis of the complete mitochondrial genome of *Hyla suweonensis* (Anura: Hylidae)

**DOI:** 10.1080/23802359.2017.1292475

**Published:** 2017-02-23

**Authors:** Mu-Yeong Lee, Hey Sook Jeon, Mi-Sook Min, Junghwa An

**Affiliations:** a Animal Resources Division, National Institute of Biological Resources, Incheon, Republic of Korea;; b Conservation Genome Resources Bank for Korean Wildlife (CGRB) and Research Institute for Veterinary Science College of Veterinary Medicine, Seoul National University, Seoul, Republic of Korea

**Keywords:** *Hyla suweonensis*, complete, mitogenome, Hylinae

## Abstract

The present study reports the complete mitochondrial genome of the Suwon tree frog *Hyla suweonensis* from South Korea. This endangered species is endemic to Korea. The circular mitogenome of *H. suweonensis* includes 16,895 bp length and contains 13 protein-coding genes, 2 ribosomal RNA genes, 22 transfer RNA genes, and a non-coding region, which is the typical gene arrangement found in the available Hylinae mitogenomes. Phylogenetic analysis of six *Hyla* spp. mitochondrial genomes revealed that *H. suweonensis* is closer to *H. tsinlingensis*.

The Suweon tree frog *Hyla suweonensis*, family Hylidae, is a tree frog endemic to Korea. The distribution of this species is limited, as it is only found in Incheon, Gyeonggi-, Chungcheong-, and Jeollabuk-do in South Korea, and its typical habitats are rice paddies and wetlands (Yang et al. 1997; Lee & Park 2016). Since *H. suweonensis* first description in 1980 (Kuramoto 1980), much of its historic habitat disappeared and it now depends on anthropogenically altered habitats (Borzée & Jang 2015), and in 2012 it was listed as an endangered species I by the Ministry of Environment of Korea. Chun et al. (2012) examined the genetic diversity of Suweon tree frogs based on two fragments of mitochondrial DNA (cytochrome *b* and cytochrome oxidase subunit I genes), which revealed a low level of genetic diversity and restricted gene flow. In the present study, the complete mitogenome of the Suweon tree frog was sequenced and compared with available *Hyla* spp. mitogenomes. In addition, phylogenetic analysis based on these mitogenomes was carried out to evaluate the phylogenetic position of *H. suweonensis* within the genus.

The *H. suweonensis* (IN589) used in the present study was collected from Pyeongtaek-si, Gyeonggi-do, South Korea and deposited in the National Institute of Biological Resources (NIBR) at Incheon, South Korea. Total genomic DNA was isolated using the DNeasy Blood & Tissue kit (Qiagen, Valencia, CA) according to the manufacturer’s protocol, and the complete mitogenome sequence was determined using the primer-walking approach. Raw sequences were assembled, edited, and corrected by eye in Geneious 8.1.9 (Kearse et al. 2012). Protein-coding genes (PCGs) and ribosomal RNAs (rRNAs) were identified using DOGMA WebServer (Wyman et al. 2004) and AIRWIN (Laslett & Canbäck 2008).

The complete mitochondrial genome of *H. suweonensis* was 16,895 bp in length (GenBank accession KY419887), and consisted of 13 typical vertebrate PCGs, 22 transfer RNA genes, 2 rRNA genes (for the small and large subunits, i.e. *rrnS* and *rrnL*, respectively), and a putative control (D-loop) region, agreeing with the typical vertebrate gene arrangement (Anderson et al. 1981). Most of these genes were encoded on the H-Strand, with the exceptions of the *ND6* gene and eight tRNAs that were encoded on the L-stand. Overall, *H. suweonensis* mitogenome comprised 29.2% A, 27.4% C, 14.9% G, and 28.5% T. The putative D-loop region had two tandem repeat areas, which were 160 and 121 bp in length.

The phylogenetic relationships of the six available *Hyla* spp. mitogenomes were analyzed using the neighbour-joining (NJ) method in MEGA 6 (Tamura et al. 2013), based on 13 concatenated PCGs. This phylogenetic reconstruction showed that *H. suweonensis* is closer to *H. tsinlingensis*, contradicting previous studies (Hua et al. 2009; Duellman et al. 2016) (Figure 1). The results provided here are fundamental resources for resolving Suweon tree frogs’ phylogenetic issues and for population genetic studies aiming their conservation.

**Figure 1.**
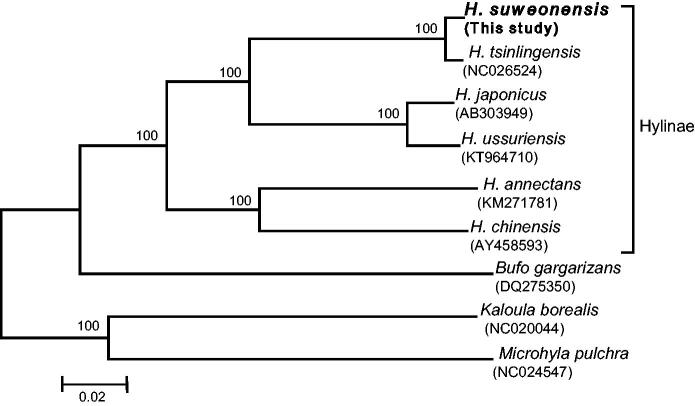
Neighbour-joining (NJ) phylogenetic tree of six *Hyla* spp. frogs based on the concatenated nucleotide sequences of 13 mitochondrial protein-coding genes. Numbers at each node represent the bootstrap support value of the NJ analysis, based on 1000 replicates, and numbers below species names indicate their GenBank accession code.
